# Methods to Quantify Soft Tissue–Based Cranial Growth and Treatment Outcomes in Children: A Systematic Review

**DOI:** 10.1371/journal.pone.0089602

**Published:** 2014-02-27

**Authors:** Sander Brons, Machteld E. van Beusichem, Ewald M. Bronkhorst, Jos M. Draaisma, Stefaan J. Bergé, Jan G. Schols, Anne Marie Kuijpers-Jagtman

**Affiliations:** 1 Department of Orthodontics and Craniofacial Biology, Radboud University Nijmegen Medical Centre, Nijmegen, The Netherlands; 2 Department of Preventive and Curative Dentistry, Radboud University Nijmegen Medical Centre, Nijmegen, The Netherlands; 3 Department of Pediatrics, Radboud University Nijmegen Medical Centre, Nijmegen, The Netherlands; 4 Department of Oral and Maxillofacial Surgery, Radboud University Nijmegen Medical Centre, Nijmegen, The Netherlands; Harvard Medical School, United States of America

## Abstract

**Context:**

Longitudinal assessment of cranial dimensions of growing children provides healthcare professionals with information about normal and deviating growth as well as treatment outcome.

**Objective:**

To give an overview of soft tissue–based methods for quantitative longitudinal assessment of cranial dimensions in children until age 6 years and to assess the reliability of these methods in studies with good methodological quality.

**Data source:**

PubMed, EMBASE, Cochrane Library, Web of Science, Scopus, and CINAHL were searched. A manual search was performed to check for additional relevant studies.

**Study selection:**

Primary publications on facial growth and treatment outcomes in children younger than age 6 years were included.

**Data extraction:**

Independent data extraction was performed by two observers. A quality assessment instrument was used to determine methodological quality. Methods used in studies with good methodological quality were assessed for reliability expressed as the magnitude of the measurement error and the correlation coefficient between repeated measurements.

**Results:**

In total, 165 studies were included, forming three groups of methods: head circumference anthropometry, direct anthropometry, and 2D photography and 3D imaging techniques (surface laser scanning and stereophotogrammetry). In general, the measurement error was below 2 mm, and correlation coefficients were very good.

**Conclusion:**

Various methods for measuring cranial dimensions have shown to be reliable. Stereophotogrammetry is the most versatile method for quantitative longitudinal assessment of cranial dimensions and shapes in children. However, direct anthropometry continues to be the best method for routine clinical assessments of linear cranial dimensions in growing children until age 6 years.

## Introduction

Longitudinal assessment of cranial dimensions of growing children provides healthcare professionals with information about normal and deviating growth as well as treatment outcome, for example in cases of deformational plagiocephaly and craniosynostosis [1], [2]. Accurate quantitative evaluation of cranial dimensions by comparison of an individual patient to normative values can provide insight into an underlying pathologic process or create a basis for treatment planning, as in cases of autism and hydrocephalus [3], [4].

Various methods for quantitative evaluation of craniofacial dimensions have been described for a variety of purposes. The standard technique is direct anthropometry, which has been extensively used for the study of craniofacial dimensions in the past century [5]. Two-dimensional (2D) x-ray cephalometry [6]–[8] and photography [9], [10] also have been applied for decades and even today are the most commonly used records for dento-skeletal and facial diagnosis. Recent technological advancements have led craniofacial researchers and clinicians into the era of three dimensional (3D) digital imaging. Techniques like cone beam computed tomography [11], [12], surface laser scanning [13], [14], and stereophotogrammetry [15]–[17] have become available for describing and comparing craniofacial dimensions and shapes, making a diagnosis or planning treatment, and evaluating growth and treatment outcomes.

In an earlier systematic review, we described various methods for quantitative evaluation of facial dimensions in children up to age 6 years for a variety of purposes [18]. This study describes the methods for quantitative evaluation of cranial dimensions. Its aims are to 1) give an overview of soft tissue–based methods for quantitative longitudinal assessment of cranial dimensions in children up to age 6 years; 2) assess the methodological quality of the studies using such approaches; and 3) assess the reliability of these methods applied in studies with good methodological quality.

## Methods

### Protocol and registration

Inclusion criteria and methods of analysis were specified in advance and documented in a protocol. A registration number is not available for this review since PROSPERO [19] was still in development when it was performed.

### Eligibility criteria

Eligible for inclusion were primary publications reporting on 1) soft tissue–based evaluation of the head and face; 2) children under age 6 years at the start of the study; 3) quantitative changes; and 4) longitudinal studies.

Excluded were publications describing 1) skeletal changes, 2) fetal growth, 3) animal studies, or 4) cross-sectional studies or featuring 5) case reports, reviews, and letters. No restrictions for language, publication date, and publication status were imposed.

### Information resources

Studies were identified by searching electronic databases. The search was applied to PubMed (from 1948), EMBASE Excerpta Medica (from 1980), Cochrane Library (from 1993), Web of Science (from 1945), Scopus (from 2004), and CINAHL (from 1982). The last search was run on October 1, 2012. In addition, we manually searched the reference lists of included studies for potentially eligible studies. Digital full-text publications were retrieved from licensed digital publishers, and paper publications were retrieved from the library. In cases in which the full-text publication could not be retrieved, authors were requested by e-mail to provide the article. The gray literature was not searched.

### Search strategy

The search strategy was developed and databases selected with the help of a senior librarian specialized in health sciences. Databases selected were PubMed, EMBASE, Excerpta Medica, Cochrane Library, Web of Science, Scopus, and CINAHL. Medical Subject Headings and free-text words were used for the search strategy of PubMed (Table 1). The search strategies for the other databases were directly derived from the former. The last search was performed on October 1, 2012.

**Table 1:** Search strategy PubMed.

Search strategy PubMed
(“Face”[Mesh:noexp] OR face[TiAb] OR facial[TiAb] OR craniofacial[TiAb] OR OR OR born*
craniomaxillofacial[TiAB] OR maxillofacial[TiAb] OR dentofacial[TiAb] OR “Facies”[Mesh]
facies[TiAb] OR “Head”[Mesh:noexp] OR head[TiAb]) AND (“Growth and
Development”[Mesh:noexp] OR “Growth”[Mesh:noexp] OR “growth and development”[Sh]
growth[TiAb] OR “Anthropometry”[Mesh:noexp] OR anthropometr*[TiAb] OR
“cephalometry”[Mesh] OR cephalometr*[TiAb] OR “imaging, three-dimensional”[MeSH
Terms] OR “three-dimensional imaging”[TiAb] OR “3d imaging”[TiAb] OR
“Photogrammetry”[Mesh] OR photogrammetry[TiAb] OR “Tomography, X Ray
Computed”[Mesh] OR “Tomography, X Ray Computed”[TiAb] OR “Lasers”[Mesh:noexp] OR
laser[TiAb] OR “Magnetic Resonance Imaging”[Mesh:noexp] OR “magnetic resonance
imaging”[TiAb] OR MRI[TiAb]) AND (infant OR infan* OR newborn OR newborn* OR new
OR baby OR baby* OR babies OR neonat* OR perinat* OR postnat* OR toddler* OR
kindergar* OR preschool* OR pre school) AND (“Cohort Studies”[Mesh] OR ((cohort[TiAb]
OR longitudinal[TiAb] OR followup[TiAb] OR follow up*[TiAb]) AND (study[TiAb] OR
studies[TiAb])))

The search strategy focused on four categories of terms, as follows: (1) terms to search for the population of interest (*i.e*., babies, infants, and pre-school children), for which a selection of the appropriate terms from the ‘Child’ search strategy was made to sort out citations not reporting on children between 0 and 6 years of age [20]; (2) terms to search for growth and methods for quantitative evaluation (*i.e.*, growth, anthropometrics, and imaging techniques); (3) terms to search for the anatomic region of interest (*i.e.*, face and head); and (4) terms to search for the longitudinal aspect (*i.e.*, cohort and follow-up studies).

### Study selection

First, studies were independently screened on title and abstract by two reviewers (SB and MB) in a standardized manner. In an additional step, disagreements between reviewers were resolved by discussion and consensus. Second, full-text assessments for eligibility were independently performed by the same two reviewers in a standardized manner. Again, in an additional step, disagreements were resolved by discussion and consensus. Results of both first and second independently performed assessments of eligibility were analyzed to assess inter-rater reliability. Third, the first author performed a manual search of the reference lists of the included studies. Finally, all included studies were categorized as describing facial or cranial evaluation of growth and treatment outcome. The plane connecting the glabella with the left and right euryon arbitrarily separates the cranium from the face. Measurements on or above this plane were considered to be cranial and those below this plane to be facial. Studies describing cranial evaluation of growth and treatment are included in this review.

### Quality assessment

Study quality was assessed by the quality assessment instrument (QAI) for clinical trials used by Gordon et al. (Table 2) [21]. This instrument includes an assessment of study bias. A checkmark was made when a criterion was fulfilled. Depending on study design, quality assessment was performed on a maximum of 15 criteria. In case criteria were not applicable to a certain study design, fewer than 15 criteria were scored. Study quality is expressed as the percentage of criteria fulfilled in relation to the total number of applicable criteria.

**Table 2:** Quality assessment instrument [21].

I. Study design (7 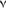 )
A. Objective—objective clearly formulated ( 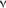 )
B. Sample size—considered adequate ( 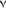 )
C. Sample size—estimated before collection of data ( 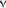 )
D. Selection criteria—clearly described ( 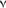 )
E. Baseline characteristics—similar baseline characteristics ( 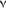 )
F. Timing—prospective ( 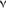 )
G. Randomization—stated ( 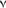 )

The score per study is calculated as a percentage by dividing the number of checkmarks by the number of applicable criteria and multiplying by 100. Studies were grouped according to similarity of methods for measurement of cranial growth or treatment outcome. A mean quality score for each group of methods was calculated. Arbitrarily, a cut-off of 60% or higher was graded as good quality and below 60% as poor quality. To assess the inter-rater reliability of the assessment of study quality, 19 randomly selected studies were scored by two reviewers (SB and AK).

### Data extraction

Methods used in studies with good methodological quality were assessed for reliability expressed as the magnitude of the measurement error and the correlation coefficient between repeated measurements.

### Statistics

Cohen's kappa statistics were used to assess the inter-rater agreement for the process of study selection and for each criterion of the QAI. According to Landis and Koch, the level of inter-rater agreement is very good if the value of K is 0.81–1.00, good if K is 0.61–0.80, moderate if K is 0.41–0.60, fair if K is 0.21–0.40, and poor if K is ≤0.20 [22].

Analysis of variance and non-parametric Kruskal-Wallis tests were performed to evaluate differences in mean scores between groups of methods. SPSS version 21.0 was used for analysis.

## Results

### Study selection

The inter-rater kappa for screening on title and abstract was 0.76. For full-text assessment of eligibility, the kappa was 0.69. The reliability of both steps in the process of study selection is qualified as good [22].

**Figure 1:**
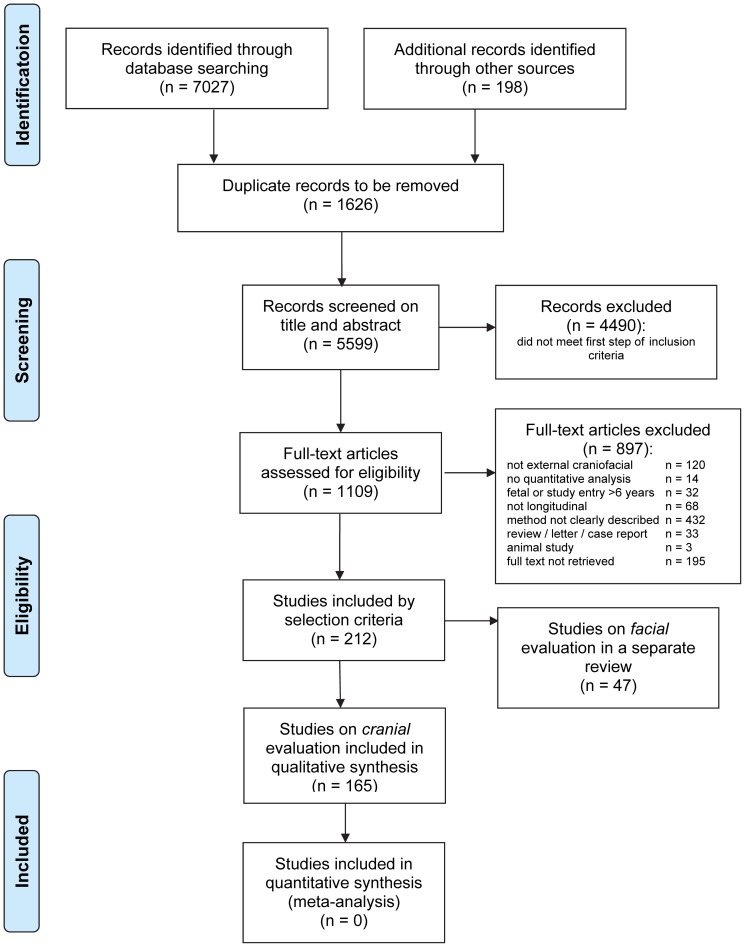
PRISMA flow diagram of the study selection process.

The search of PubMed, EMBASE, Cochrane Library, Web of Science, Scopus, and CINAHL provided a total of 7027 citations, and the manual search provided 198 citations. After adjusting for duplicates, 5599 citations remained for screening of title and abstract. Of these, 4490 studies were discarded because they did not meet the eligibility criteria so that a total of 1109 studies remained for full-text assessment. Of these, 897 studies were excluded for different reasons, and 195 were discarded because the full-text publication could not be retrieved. We ultimately identified 212 studies that met the inclusion criteria. The last step in the inclusion process divided the studies into those on facial evaluation (n = 47) and studies on cranial evaluation (n = 165). A total of 188 studies originated from the electronic databases; the remaining 24 studies originated from the additional manual search of the references of the included studies. Figure 1 shows the PRISMA flow diagram, and Checklist S1 shows the PRISMA checklist [23]. The current systematic review is restricted to studies on cranial evaluation of growth and treatment outcomes in children; a systematic review of studies on facial evaluation of growth and treatment outcomes in children is described in a separate publication [17];

Of the 165 included studies, 136 studies used direct anthropometry for head circumference, 19 studies used other direct cranial anthropometry approaches, 3 studies used 2D photography, and 7 studies used 3D imaging techniques (5 stereophotogrammetry and 2 surface laser scanning).

### Study quality assessment

Inter-rater reliability values for all 15 criteria of the QAI were between kappa 0.19 and 1.00; 11 out of 15 criteria had a kappa of 0.50 or higher. Inter-rater agreement on criteria E (similar baseline characteristics), I (blind measurement), and K (dropouts included in data analysis) was below 0.20.

Assessment of methodological quality of all reviewed studies resulted in scores ranging from 20% to 100%. A total of 118 studies qualified as good based on a methodological quality score equal to or above 60%. Score summaries of studies with good methodological quality using direct anthropometry for head circumference [24]–[118] are shown in Table 3 (n = 95); those for other direct anthropometry [119]–[132] are shown in Table 4 (n = 14); and those for indirect 2D and 3D imaging techniques [133]–[141] are shown in Table 5 (n = 9).

**Table 3:** Methodological quality scores of studies using direct anthropometry for head circumference reporting on soft tissue–based quantitative longitudinal assessment of cranial dimensions in children until age 6 years with a score equal to or above 60% (n = 95) [24]–[118].

First author	Year	Design							Measure			Statistics					Score
		A	B	C	D	E	F	G	H	I	J	K	L	M	N	O	
Head circumference anthropometry																	
Belfort	2012	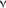	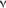	o	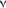	.	o	.	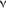	.	o	.	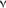	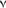	o	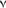	64%
Bendersky	1998	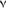	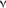	o	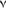	.	o	.	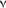	.	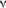	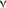	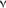	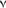	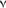	o	75%
Berry	1997	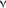	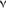	o	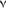	.	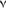	.	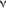	.	o	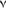	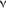	.	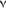	o	64%
Bhalla	1993	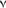	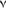	o	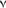	.	o	.	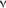	.	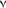	.	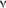	.	o	o	60%
Binns	1996	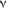	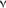	o	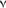	.	o	.	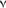	.	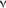	.	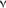	.	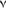	o	70%
Bouthoorn	2012	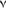	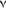	o	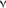	.	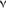	.	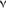	.	o	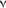	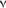	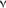	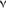	o	75%
Bracewell	2007	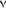	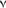	o	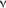	.	o	.	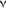	o	o	.	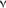	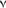	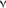	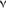	67%
Butz	2005	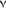	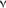	o	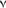	.	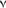	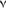	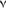	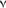	o	o	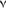	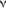	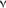	o	69%
Cardoso	2007	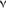	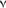	o	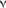	.	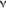	.	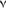	.	o	o	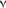	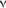	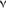	o	67%
Carlson	1996	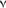	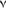	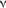	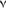	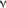	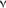	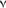	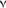	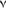	o	o	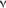	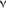	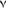	o	80%
Chaudhari	2012	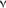	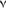	o	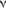	.	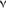	.	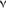	o	o	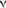	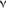	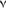	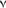	o	69%
De Bruin	1998	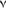	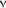	o	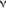	.	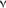	o	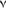	o	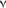	o	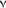	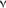	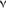	o	64%
DeReignier	1996	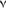	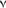	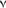	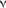	.	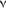	.	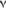	o	o	o	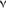	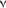	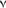	o	69%
Desmyttere	2009	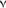	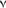	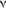	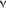	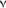	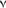	.	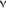	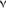	o	o	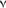	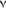	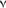	o	79%
Desmyttere	2009	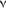	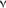	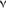	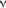	.	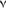	o	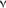	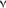	o	o	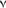	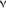	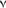	o	71%
Donma	1997	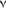	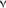	o	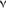	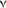	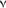	o	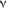	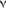	o	.	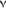	o	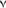	o	64%
D'Souza	1986	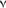	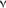	o	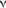	.	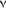	.	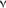	o	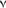	o	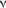	o	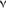	o	62%
Durmus	2011	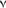	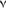	o	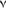	.	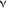	.	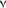	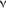	o	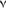	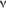	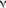	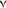	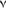	85%
Ekblad	2010	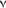	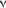	o	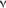	.	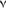	.	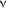	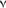	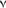	o	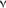	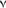	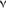	o	77%
Elwood	1987	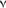	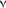	o	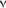	.	o	.	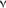	.	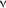	.	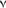	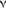	o	o	64%
Erasmus	2002	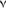	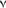	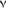	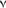	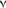	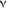	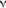	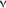	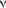	o	o	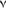	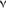	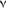	o	80%
Eregie	2001	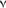	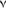	o	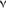	.	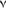	.	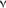	.	o	o	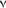	.	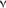	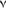	73%
Ernst	1990	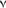	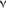	o	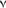	.	o	.	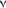	.	o	.	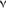	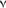	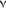	o	64%
Farooqi	2006	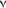	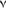	o	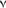	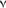	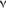	.	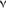	.	o	o	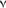	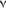	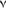	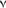	77%
Ford	2000	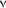	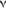	o	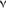	.	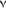	.	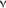	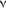	o	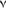	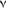	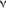	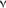	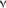	85%
Ford	1986	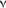	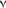	o	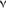	.	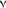	.	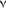	.	o	o	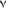	.	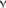	o	64%
Friel	1993	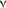	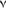	o	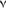	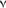	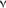	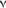	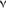	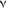	o	o	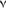	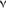	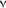	o	73%
Fukumoto	2008	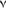	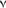	o	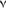	.	o	.	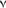	.	o	.	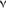	.	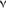	o	60%
Gale	2006	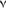	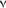	o	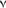	o	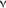	.	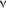	.	o	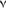	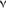	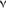	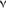	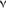	77%
Gale	2004	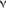	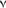	o	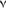	.	o	.	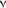	.	o	.	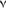	.	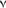	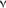	70%
Georgieff	1995	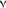	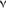	o	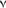	.	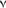	.	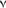	o	o	o	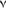	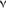	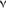	o	62%
Georgieff	1989	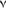	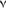	o	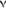	.	o	.	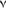	o	o	.	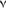	.	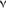	o	60%
Gross	1983	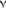	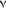	o	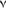	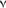	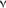	.	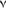	.	o	o	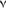	o	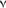	o	62%
Gross	1983	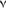	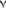	o	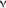	.	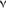	.	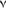	.	o	o	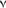	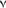	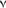	o	75%
Guo	1988	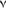	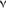	o	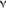	.	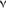	.	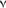	.	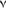	.	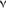	.	o	o	60%
Hagelberg	1990	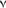	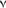	o	o	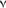	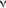	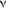	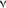	o	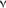	o	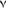	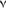	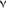	o	67%
Hansen-pupp	2011	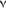	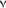	o	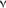	.	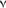	.	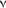	.	o	o	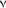	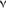	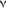	o	67%
Herrmann	2010	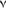	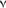	o	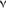	.	o	.	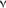	.	o	.	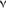	.	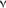	o	60%
Ishikawa	1987	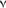	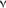	o	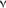	.	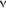	.	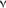	.	o	o	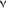	.	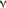	.	69%
Jaffe	1992	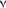	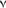	o	o	.	o	.	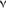	.	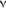	.	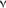	.	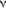	o	60%
Jaldin	2011	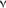	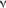	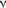	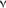	.	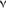	.	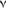	.	o	o	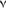	.	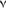	o	73%
Jaruratanasirikul	1999	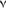	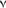	o	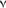	.	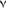	.	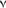	o	o	o	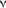	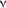	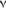	o	62%
Kan	2007	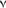	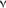	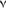	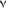	.	o	.	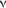	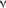	o	.	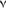	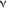	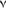	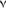	77%
Karatza	2003	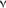	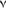	o	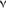	.	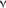	.	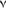	o	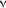	o	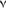	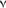	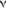	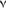	77%
Kiran	2007	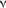	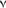	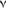	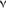	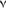	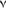	.	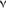	o	o	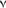	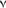	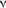	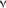	o	79%
Kitchen	1992	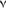	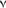	o	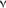	.	o	.	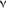	o	o	.	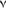	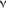	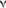	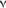	67%
Koo	2006	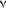	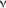	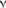	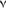	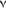	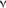	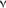	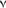	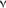	o	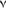	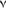	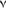	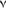	o	87%
Lainhart	1997	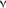	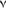	o	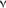	.	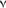	.	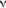	.	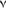	o	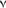	.	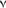	o	75%
Lasekan	2011	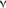	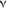	o	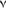	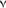	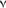	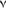	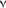	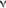	o	o	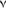	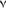	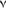	o	73%
Lasekan	2006	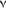	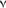	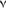	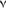	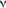	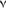	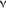	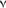	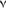	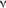	o	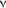	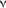	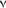	o	87%
Lira	2009	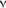	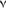	o	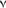	.	o	.	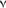	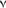	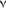	.	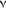	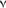	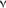	o	75%
Lucas	2001	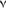	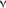	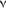	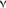	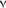	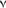	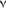	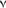	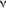	o	o	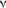	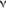	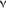	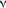	87%
Maguire	2008	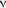	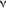	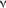	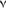	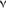	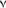	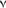	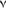	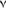	o	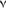	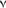	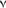	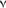	o	87%
Makrides	2000	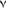	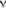	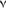	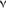	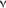	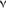	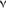	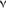	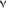	o	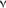	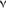	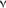	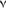	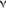	93%
Makrides	1999	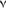	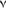	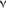	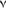	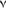	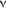	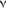	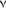	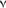	o	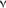	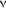	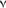	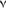	o	87%
Mamabolo	2004	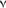	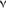	o	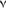	.	o	.	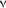	.	o	.	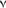	.	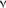	o	60%
Marks	1979	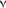	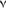	o	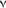	.	o	.	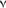	.	o	.	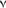	.	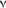	o	60%
Maserei	2007	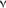	o	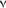	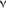	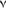	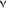	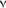	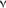	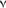	o	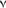	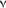	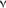	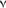	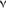	87%
Mathur	2009	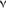	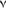	o	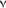	.	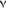	.	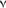	.	o	o	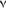	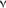	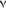	o	62%
McCowan	1999	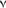	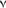	o	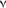	.	o	.	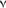	.	o	.	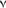	.	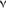	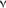	70%
McLeod	2011	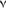	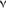	o	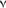	.	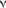	.	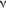	.	o	o	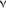	.	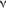	o	64%
Mercuri	2000	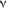	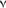	o	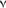	.	o	.	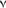	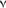	o	.	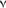	.	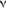	o	64%
Meyer-Marcotty	2012	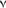	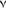	o	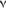	.	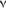	.	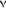	o	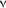	o	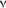	o	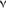	o	62%
Moore	1995	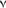	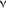	o	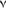	.	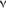	.	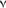	.	o	o	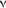	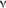	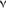	o	62%
Moye	1993	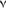	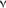	o	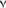	.	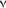	.	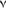	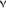	o	o	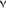	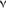	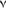	o	69%
Nelson	1997	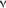	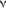	o	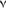	.	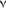	.	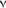	.	o	o	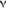	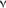	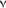	o	62%
Ochiai	2008	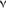	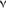	o	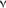	.	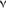	.	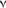	.	o	o	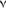	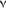	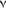	o	73%
Olivan	2003	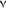	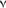	o	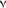	.	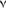	.	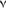	.	o	o	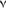	.	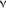	o	64%
Oliveira	2007	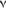	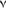	o	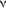	.	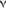	.	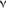	.	o	o	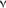	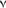	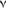	o	67%
Padilla	2010	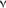	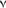	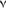	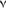	.	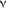	.	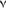	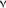	o	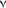	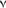	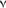	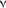	o	85%
Paul	2008	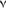	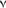	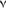	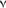	.	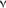	.	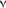	.	o	o	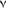	.	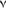	o	72%
Peng	2005	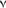	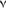	o	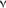	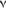	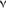	.	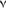	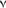	o	o	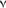	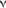	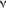	o	71%
Piemontese	2011	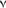	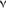	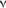	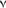	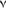	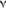	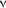	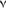	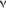	o	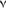	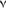	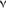	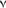	o	87%
Polberger	1999	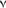	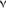	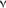	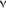	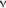	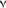	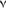	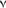	o	o	o	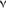	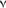	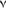	o	73%
Rijken	2007	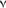	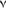	o	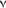	.	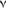	.	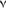	.	o	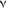	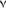	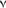	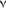	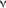	77%
Roberfroid	2012	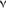	o	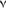	o	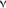	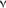	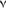	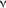	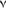	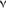	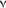	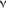	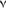	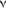	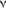	87%
Roche	1987	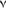	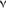	o	o	.	o	.	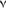	.	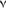	o	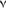	.	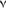	o	60%
Rodriguez Garcia	2003	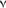	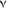	o	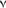	.	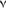	.	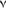	.	o	.	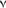	.	o	o	60%
Ross	2012	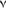	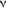	o	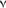	.	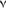	.	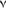	.	o	o	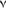	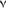	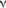	o	67%
Rothenberg	1999	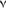	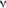	o	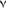	.	o	.	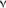	.	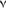	.	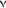	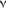	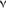	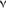	82%
Saliba	1990	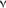	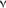	o	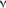	.	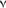	.	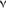	o	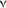	o	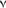	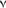	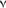	o	69%
Sawada	2010	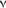	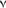	o	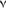	.	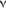	.	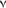	.	o	o	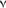	.	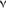	o	64%
Schaefer	1994	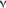	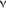	o	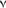	.	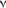	.	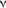	.	o	o	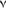	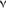	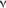	o	62%
Sharma	2011	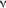	o	o	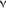	.	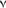	.	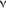	.	o	o	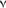	.	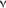	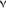	64%
Shaw	1999	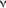	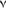	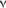	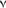	o	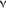	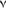	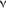	o	o	o	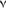	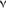	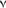	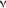	76%
Sheth	1995	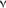	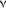	o	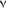	.	o	.	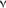	.	o	.	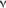	o	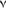	o	60%
Shortland	1998	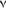	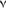	o	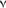	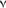	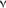	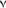	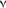	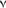	o	o	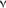	o	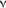	o	76%
Tan	2008	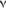	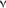	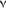	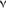	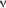	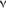	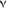	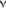	o	o	o	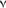	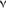	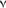	o	73%
Tinoco	2009	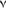	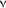	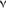	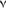	.	o	.	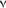	.	o	.	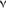	.	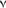	o	70%
Touwslager	2008	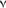	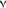	o	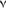	.	o	.	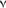	.	o	.	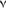	.	o	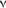	60%
Vaidya	2008	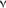	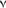	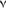	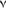	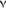	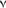	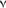	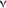	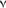	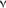	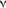	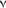	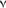	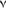	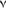	100%
Van Daalen	2007	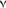	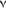	o	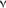	.	o	.	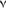	.	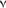	.	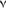	.	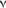	o	70%
Whitehouse	2010	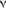	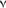	o	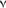	.	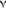	.	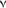	.	o	o	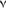	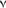	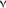	o	67%
Wood	2003	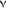	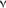	o	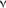	.	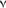	.	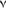	.	o	o	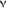	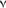	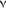	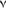	75%
Zabaneh	2011	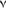	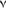	o	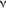	.	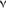	.	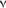	.	o	o	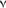	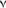	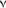	o	67%

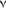
  =  fulfilled the methodological criteria satisfactorily.

o  =  did not fulfill the methodological criteria.

.  =  not applicable.

**Table 4:** Methodological quality scores of studies using direct anthropometry reporting on soft tissue–based quantitative longitudinal assessment of cranial dimensions in children until age 6 years with a score equal to or above 60% (n = 14) [119]–[132].

First author	Year	Design							Measure			Statistics					Score
		A	B	C	D	E	F	G	H	I	J	K	L	M	N	O	
Anthropometry																	
Agrawal	2006	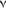	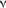	o	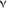	.	o	.	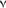	.	o	.	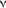	.	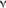	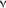	70%
Chatterjee	2009	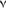	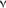	o	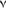	.	o	.	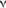	.	o	.	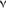	.	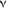	o	60%
Fearon	2009	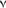	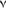	o	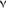	.	o	.	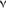	.	o	.	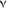	.	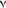	o	60%
Fearon	2006	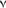	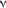	o	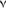	.	o	.	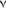	.	o	o	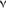	.	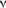	o	60%
Kelly	1999	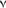	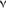	o	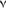	.	o	.	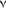	.	o	.	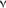	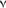	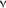	o	64%
Lee	2006	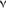	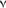	o	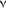	.	o	.	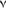	.	o	.	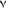	.	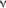	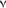	70%
Lee	2008	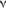	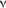	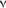	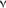	.	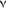	.	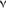	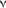	o	o	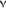	.	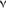	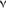	77%
Littlefield	1998	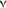	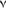	o	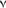	.	o	.	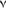	.	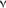	.	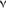	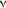	o	o	64%
Mulliken	1999	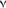	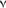	o	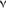	.	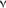	.	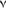	o	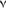	.	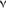	o	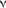	o	62%
Pedroso	2008	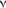	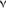	o	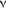	.	o	.	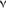	.	o	.	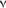	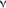	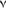	o	64%
Teichgraeber	2004	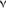	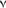	o	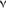	.	o	.	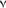	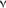	o	.	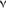	.	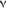	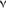	62%
Teichgraeber	2002	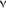	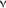	o	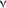	.	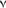	.	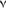	.	o	o	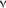	.	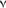	o	64%
Van Vlimmeren	2008	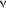	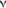	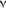	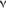	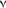	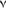	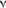	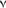	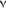	o	o	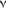	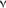	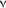	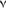	78%
Van Vlimmeren	2007	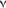	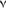	o	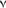	.	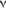	.	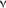	o	o	o	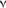	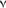	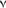	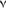	69%

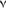
  =  fulfilled the methodological criteria satisfactorily.

o  =  did not fulfill the methodological criteria.

.  =  not applicable.

**Table 5:** Methodological quality scores of studies using indirect 2D and 3D imaging techniques reporting on soft tissue–based quantitative longitudinal assessment of cranial dimensions in children until age 6 years with a score equal to or above 60% (n = 9) [133]–[141].

First author	Year	Design							Measure			Statistics					Score
		A	B	C	D	E	F	G	H	I	J	K	L	M	N	O	
2D photography																	
Hutchinson	2010	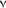	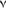	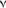	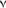	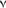	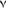	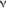	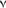	o	o	o	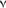	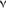	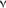	o	73%
Hutchinson	2004	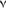	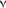	o	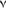	.	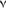	.	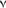	.	o	o	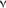	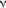	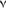	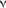	75%
Hutchison	2011	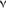	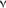	o	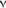	.	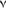	.	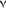	.	o	o	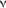	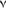	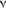	o	67%
3D surface laser scanning																	
Plank	2006	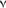	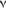	o	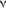	.	o	o	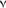	o	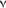	.	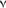	o	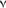	o	62%
3D stereophotogrammetry																	
Collet	2012	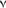	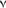	o	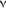	.	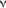	.	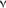	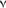	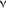	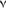	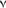	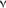	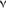	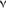	92%
Lipira	2010	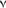	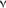	o	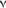	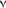	o	.	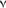	o	o	.	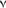	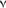	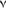	o	62%
Meyer-Marcotty	2012	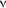	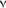	o	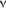	.	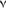	.	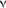	o	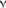	o	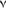	o	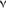	o	62%
Schaaf	2010	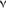	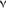	o	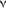	.	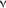	.	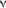	.	o	o	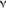	.	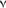	o	64%
Toma	2010	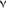	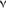	o	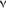	.	o	.	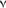	.	o	.	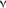	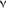	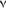	o	64%

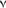
  =  fulfilled the methodological criteria satisfactorily.

o  =  did not fulfill the methodological criteria.

.  =  not applicable.

Analysis of variance (p = 0.14) and Kruskal-Wallis test (p = 0.16) revealed no statistically significant difference for methodological quality, expressed as a percentage between groups of methods.

### Reliability

Scores for reliability of methods for soft tissue–based quantitative longitudinal assessment are shown in Table 6. Only 12 of the 118 studies with good methodological quality report data for the reliability of the method to quantify cranial dimensions; 8 studies used direct anthropometry for head circumference [25], [27], [35], [58], [63], [71], [100], [103], two used other kinds of direct anthropometry [126], [127], one study used 3D laser scanning [136], and one used 3D stereophotogrammetry [139].

**Table 6:** Reliability of methods for soft tissue–based quantitative longitudinal assessment of cranial dimensions in children until age 6 years in studies with good methodological quality (methodological quality score equal to or above 60%).

First author	Year	Measurement error	Correlation coefficient
Head circumference, direct anthropometry			
Bendersky	1998	.	0.99
Bhalla	1993	0–0.2 mm	.
De Bruin	1998	ns	.
Guo	1988	1.6 mm	.
Jaffe	1992	1–2 mm	.
Lainhart	1997	.	0.87
Roche	1987	0.9 mm	.
Rothenberg	1999	0.8–1.7 mm	.
Direct anthropometry			
Littlefield	1998	1 mm	.
Mulliken	1999	1 mm	.
3D laser scanning			
Plank	2006	0.5 mm	.
3D stereophotogrammetry			
Meyer-Marcotty	2012	0.02–4.3 mm	.

.  =  not reported.

ns  =  not significant.

Regarding direct anthropometry for head circumference, 5 studies with good methodological quality reported a measurement error equal to or below 1.7 mm. Two studies with good methodological quality using direct anthropometry reported correlation coefficients between repeated measurements of 0.87 and 0.99 and were both qualified as very good. Regarding other kinds of direct anthropometry, two studies with good methodological quality reported a measurement error of 1 mm.

Studies of good methodological quality using 2D photography and reporting the measurement error or correlation coefficients were not identified among the included studies. One study with good methodological quality using 3D laser scanning reported a measurement error of 0.5 mm, and another using 3D stereophotogrammetry reported a measurement error of 0.02–4.3 mm.

## Discussion

### Summary of evidence

The objectives of this systematic review were to 1) give an overview of soft tissue–based methods for quantitative longitudinal assessment of cranial dimensions in children until age 6 years; 2) assess the methodological quality of the studies using such approaches; and 3) assess reliability of these quantitative measurement methods used in studies with good methodological quality.

In the literature, various terms to describe measurement error exist. Some studies use accuracy to describe landmark identification error, which in turn may consist of operator error, capture error, and registration error [142]. More often in the literature, reliability is used to describe landmark identification error of a method. Reliability can be expressed by the measurement error or a correlation coefficient between repeated measurements [9], [143], [144] and represents the ability of observers to make a consistent analysis. In this systematic review, reliability in studies with good methodological quality was assessed and expressed by duplicate measurement errors and correlation coefficients between repeated measurements. Direct measurement of head circumference is the most often used method for soft tissue–based evaluation of cranial growth and treatment outcomes. The use of growth charts in the clinical assessment of growing infants and children and in pediatric nutritional screening and epidemiologic assessments has already been recommended for decades [27]. For this purpose, length and weight also are recorded in many countries from birth onwards. Direct anthropometry for head circumference seems to be a generally accepted method for most researchers because only 8 out of 95 studies with good methodological quality reported on its reliability. Measurement errors varied from 0.2 to 1.7 mm, and correlation coefficients were very good. Other kinds of direct anthropometry yielded a measurement error of 1 mm. Normal growth of head circumference shows an increase for mean head circumference from 34–36 cm at birth to 51–52 by age 6 years [145]. The measurement errors for head circumference anthropometry presented in this review are within 1% of the values of normal growth, which seems to be negligible. Direct anthropometry is a reliable and cheap method to study linear dimensions and has been regarded as the gold standard for many decades, but it requires patient cooperation and precludes archiving [146].

Photographic techniques, on the other hand, can capture images for data storage. The most common imaging technique is 2D photography, which has the advantages of being safe, relatively cheap, and user friendly. However, because none of the included studies reported a measurement error or correlation coefficient, its reliability for evaluation of cranial growth and treatment outcome is uncertain.

Various 3D imaging techniques have been recently introduced and were applied in 6 out of 165 studies included in this systematic review. Only two studies with good methodological quality reported the measurement error (Table 6). The measurement error in one study using 3D laser scanning was 0.5 mm; in one study using 3D stereophotogrammetry, it was 0.02 mm for head width, 0.04 mm for head circumference, and 4.3 mm for vertex height. Therefore, 3D imaging might be a reliable method to quantify cranial dimensions. Reliability of measurements from 3D imaging seems to be more related to the exact anatomical region of interest than to the method itself.

When reviewing the literature for this study, we found only six included studies with good methodological quality using 3D imaging to quantify soft tissue–based cranial growth and treatment outcome. The explanation is that only recently have techniques become available to capture the full 360° image needed to study cranial dimensions. Most studies using 3D imaging concern facial growth and treatment outcome because this technique has been available for two decades. Therefore, it is expected that within the next decade, more studies using 3D imaging of cranial dimensions will be published. Advantages of these 3D techniques are millisecond fast image capture, archival capabilities, a good-resolution color representation, and no exposure to ionizing radiation. Furthermore, assessment of linear dimensions and cranial size and shape can be made three-dimensionally. These are major advantages compared to more simplistic analyses performed with direct anthropometry. A shortcoming of 3D imaging is its inability to capture surface morphology in the presence of cranial hair. The reliability of 3D imaging techniques for soft tissue–based evaluation of cranial growth and treatment outcomes needs to be investigated further.

### Limitations

Failure to identify all relevant reports for a systematic review is likely to result in bias [147]. For this reason, highly sensitive search strategies were developed with the help of a senior librarian specialized in health sciences for a combination of both narrow and broad health science databases.

The process of study selection was performed in an independent standardized manner by two reviewers to prevent unjustified exclusion of eligible studies. The manual search of the reference lists of the included studies was performed by only one reviewer. Possibly eligible studies could have been missed in this stage of the selection process. However, because only approximately one out of ten studies was retrieved by the manual search, this omission might be negligible. Furthermore, every effort was made to contact the authors by email in cases where online access was not permitted or the journal was not available in the library. Nevertheless, failure to retrieve full-text publications of possibly eligible studies (n = 195) was inevitable.

The instrument used to assess methodological quality was adapted from Lagravère et al. [148] who developed a methodological quality checklist to assess study design, study measurements, and statistical analysis in clinical trials. Since the introduction the checklist has been modified and used by Gordon et al. [21] and Van Vlijmen et al. [149]. Scientific use of the checklist has been accepted because the criteria check for generally accepted reasons for bias, despite a lack of validation of the QAI. The majority of inter-rater disagreements arose in the assessment of applicability of criteria E, I, and K to certain studies (similar baseline characteristics, blind measurement, and dropouts included in data analysis, respectively). This greater frequency can be explained by the absence of adequate instructions of this QAI together with the presence of a wide variety of study designs. Therefore, raters should test this QAI thoroughly and obtain consensus before scoring. In the literature, no single tool is an obvious candidate for assessment of methodological quality of non-randomized studies [150]. Attempts to validate QAIs like the Newcastle-Ottowa [151] scale or the Jadad scale [152] produce highly arbitrary results and cannot demonstrate significant effects on quality scores [153], [154]. There is a need for a validated QAI that is preferably applicable to a wide range of study designs.

## Conclusions

Direct anthropometrical measurement of head circumference in growing children below age 6 years is a reliable method for assessing cranial dimensions. The non-invasive 3D surface laser scanning and 3D-stereophogrammetry techniques can assess size and shape three-dimensionally. However, their reliability for assessing cranial dimensions needs to be investigated further.

## Supporting Information

Checklist S1 PRISMA checklist.
(DOCX)
